# *WFSuite*: a Python software suite for X-ray wavefront sensing and at-wavelength metrology

**DOI:** 10.1107/S160057752600500X

**Published:** 2026-06-16

**Authors:** Xianbo Shi, Luca Rebuffi, Zhi Qiao, Yu-Chung Lin, Lahsen Assoufid

**Affiliations:** a Advanced Photon Source Argonne National Laboratory 9700 S. Cass Ave Lemont IL60439 USA; RIKEN SPring-8 Center, Japan

**Keywords:** X-ray wavefront sensing, at-wavelength metrology, coded mask, speckle tracking

## Abstract

*WFSuite* is a Python-based software suite for X-ray wavefront sensing and at-wavelength metrology for synchrotron and X-ray free-electron laser applications. It integrates tools for both relative and absolute speckle-based measurements. In the relative metrology module, *WFSuite* implements wavelet-transform-based X-ray speckle tracking (WXST) and wavelet-transform-based speckle vector tracking (WSVT) methods to retrieve differential phase and wavefront distortions by comparing speckle patterns recorded with and without the test sample. In the absolute phase module, *WFSuite* uses coded-mask speckle patterns and replaces the measured reference with a numerically simulated one, enabling single-shot wavefront reconstruction using either WXST or a neural-network-based method (SPINNet). The suite supports 2D image inputs in TIFF and HDF5 formats and outputs displacement fields, phase gradients, and reconstructed wavefronts. *WFSuite* has been deployed at several synchrotron beamlines for optics characterization, beam diagnostics, and real-time alignment. Its modular design and unified interface provide a flexible and extensible framework for routine wavefront analysis and integration with beamline control systems.

## Abstract 2

*WFSuite* is an open-source Python suite for speckle-based X-ray wavefront reconstruction, offering both relative metrology and absolute phase modules. It streamlines wavefront characterization and feedback control for synchrotron and XFEL applications.

## 1. Introduction

Modern synchrotron and X-ray free-electron laser facilities place increasingly stringent demands on beam quality and stability. High-coherence sources, advanced focusing optics, and complex experimental geometries all require precise knowledge and control of the X-ray wavefront at the sample position. At-wavelength wavefront sensing and optics metrology have therefore become essential tools for commissioning new beamlines, diagnosing optics imperfections, and maintaining reliable beam delivery during user operations (Cocco *et al.*, 2022 ▸).

A variety of X-ray wavefront sensing techniques have been developed over the past decade, including grating interferometry (Grizolli *et al.*, 2017 ▸; Matsuyama *et al.*, 2012 ▸; Liu *et al.*, 2018 ▸), ptychography (Kewish *et al.*, 2010 ▸), Hartmann-type sensors (Idir *et al.*, 2010 ▸), and speckle-based approaches (Berujon *et al.*, 2020*a* ▸; Berujon *et al.*, 2020*b* ▸). Among these, speckle-based methods are desirable because they can be implemented with a simple setup and are compatible with a wide range of beam energies and configurations. By tracking the displacement of speckle patterns between a reference and a perturbed state, these methods provide access to wavefront phase gradients and, after integration, the full wavefront phase. Speckle tracking using purely random pattern generators, such as sandpapers or membrane filters, can only operate in a relative mode, where beam-path changes from optics or sample variations are measured with respect to an experimentally recorded reference state. Recently, we have developed a series of coded-mask-based methods (Qiao *et al.*, 2021 ▸; Frith *et al.*, 2023 ▸; Qiao, Shi, Celestre *et al.*, 2020 ▸; Qiao *et al.*, 2022 ▸; Qiao *et al.*, 2020 ▸) that not only enhance speckle contrast, enabling more robust and accurate relative-mode algorithms, but also permit absolute wavefront sensing, in which the reference pattern can be replaced by a numerically simulated one derived from the known mask design. These developments significantly broaden the capabilities of speckle-based wavefront sensing, but their practical adoption at synchrotron facilities has often relied on custom, beamline-specific scripts and analysis pipelines.

To address these needs, we have developed *WFSuite*, a Python-based software suite for X-ray wavefront sensing and at-wavelength metrology. *WFSuite* integrates multiple speckle-based algorithms within a common interface, supporting both relative and absolute wavefront measurement. The relative metrology module implements wavelet-transform-based X-ray speckle tracking (WXST) (Qiao *et al.*, 2020 ▸) and wavelet-transform-based speckle vector tracking (WSVT) (Qiao, Shi, Celestre *et al.*, 2020 ▸) for two-image and multi-scan analysis using speckle patterns generated by coded masks or other random modulators. The absolute phase module is specific to coded-mask configurations and enables single-shot wavefront reconstruction via WXST (Frith *et al.*, 2023 ▸; Shi *et al.*, 2025 ▸) or neural-network-based analysis using SPINNet (Qiao *et al.*, 2022 ▸; Frith *et al.*, 2023 ▸).

*WFSuite* provides both a graphical interface for interactive inspection and parameter tuning and a Python API for automated batch processing and integration with beamline control systems. The suite has been deployed and validated at multiple synchrotron beamlines, where it supports routine X-ray optics characterization, beam diagnostics, and on-the-fly wavefront analysis during alignment and commissioning. Details on software availability are provided in the *Data availability* section.

This paper presents the design and capabilities of *WFSuite*. Section 2 summarizes the underlying methods, Section 3 describes the software architecture and interface, Section 4 provides representative examples of relative and absolute measurements, and Section 5 offers concluding remarks and future development directions.

## 2. Methods and algorithms

**Figure 1:**
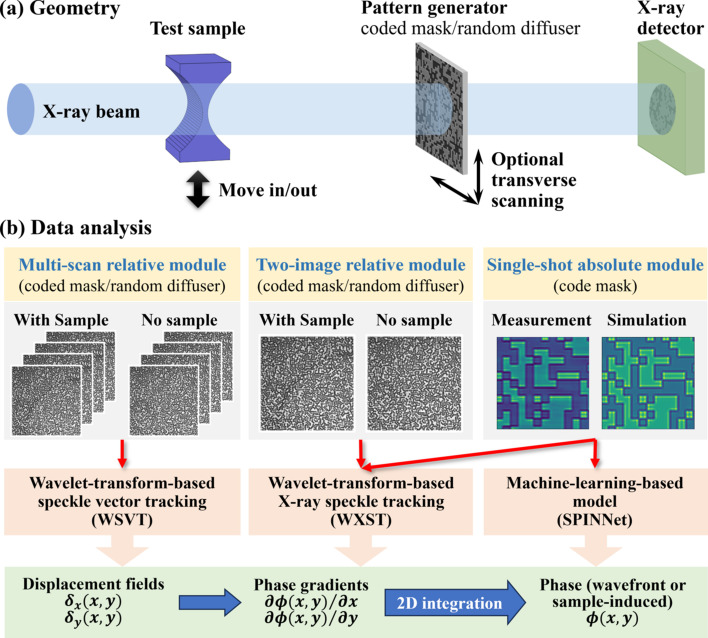
(*a*) Experimental configuration for speckle-based X-ray wavefront sensing using a coded mask or a random diffuser (*e.g.* sandpaper). (*b*) Data-analysis workflow in *WFSuite*: relative metrology module using multi-scan speckle stacks analyzed by WSVT, or two images analyzed by WXST; and absolute phase module using coded-mask single-shot measurements analyzed by WXST or SPINNet.

The overall experimental configuration and the data acquisition and analysis modules implemented in *WFSuite* are summarized in Fig. 1 ▸. The schematic in Fig. 1 ▸(*a*) illustrates how an incident X-ray beam passes through upstream optics or a sample and interacts with a speckle generator, either a coded mask or a random diffuser, whose position may be placed either upstream or downstream of the sample. The transmitted speckle pattern is then recorded by a 2D detector system.

*WFSuite* supports two categories of speckle-based wavefront sensing, as shown in Fig. 1 ▸(*b*): relative metrology and absolute phase modules. In the relative metrology module, displacement fields are retrieved by comparing two sets of speckle data acquired under different conditions. These conditions may correspond to measurements with and without the sample or optical element, or to two detector distances, or to other controlled perturbations. Relative-mode analysis can be performed using either WSVT, which operates on two multi-scan speckle stacks in which the speckle generator is translated to multiple random transverse positions, or WXST, which operates in a two-image configuration (reference + sample). The speckle generator for relative measurements may be either a random diffuser (*e.g.* sandpaper or a membrane filter) or a coded mask.

In the absolute phase module, the use of a coded mask is essential, as the known mask pattern allows the reference speckle field to be numerically simulated. Absolute-mode data can be analyzed using either WXST or SPINNet. WXST extracts displacement fields through wavelet-based speckle tracking, whereas SPINNet uses a neural-network model to infer the same quantities for subsequent phase reconstruction.

Across all modules, *WFSuite* provides the transverse displacement fields, the corresponding phase gradients, and the final reconstructed wavefront or sample-induced phase profile obtained through two-dimensional integration. In the following subsections, we summarize the three data-analysis approaches implemented in *WFSuite*: WSVT, WXST, and SPINNet, and the subsequent phase reconstruction procedures.

### 2.1. WSVT

WSVT (Qiao, Shi, Celestre *et al.*, 2020 ▸) is the multi-scan implementation of speckle vector tracking, developed for cases where the speckle generator is translated over a sequence of transverse positions to record a stack of speckle images. This multi-scan scheme produces, for every detector pixel (*x*, *y*), a one-dimensional intensity profile, *I*(*x*, *y*, *t*), where *t* denotes the scan-position index. In practice, a set of more than 40 random scan positions is typically used, covering a 2D range at least ten times larger than the characteristic speckle feature size. This ensures that the resulting profiles *I*(*x*, *y*, *t*) are sufficiently unique for each pixel, effectively serving as local fingerprints that can be tracked between measurements. When both sample and reference scans are acquired, the corresponding profiles *I*_s_(*t*) and *I*_r_(*t*) encode the modulation of the speckle pattern induced by the local beam wavefront or by sample-induced phase shifts.

To determine the displacement between the sample and reference signals, a similarity search is carried out for each pixel in the reference data over a local search window around the corresponding pixel in the sample data. WSVT performs this similarity search in the discrete wavelet transform (DWT) domain rather than through direct real-space correlation. The DWT provides a sparse, noise-robust representation of speckle intensity variations, decomposing each 1D profile into a set of approximation and detail coefficients. As the full DWT decomposition includes multiple coefficient sets (Qiao, Shi, Celestre *et al.*, 2020 ▸), we adopt the simplified notation to represent the wavelet coefficients as
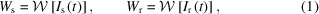
where 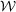 denotes the DWT operation. Because the wavelet basis is orthonormal, Euclidean distances between coefficient vectors are preserved under the transform. The transverse displacement is therefore obtained by minimizing the squared Euclidean distance between the wavelet-domain representations of the sample and reference scans,
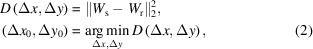
where 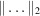 denotes the Euclidean (L2) norm.

To achieve sub-pixel accuracy, a two-dimensional paraboloid is fitted to the values of *D*(Δ*x*, Δ*y*) in a small neighborhood around the discrete minimum. The analytic minimum of this paraboloid provides a continuous estimate of (Δ*x*_0_, Δ*y*_0_), yielding smooth and precise displacement fields (δ_*x*_, δ_*y*_) even when the underlying speckle contrast is weak, or the scan step size is relatively coarse.

### 2.2. WXST

WXST (Qiao *et al.*, 2020 ▸) is a speckle-tracking method that uses two images (reference and sample) and does not require the multi-scan acquisition used in WSVT. For each detector pixel (*x*, *y*), a small two-dimensional template window is extracted from both the sample and reference images. These windows are reshaped into one-dimensional profiles, *I*_s_(*t*) and *I*_r_(*t*), where *t* indexes the pixel positions within the window. These spatial profiles serve the same role as the temporal scan profiles in WSVT, providing localized speckle signatures for displacement estimation.

After the discrete wavelet transform (DWT) is applied to the sample and reference profiles, the displacement is determined using the same coefficient-distance metric defined in Section 2.1, producing (Δ*x*_0_, Δ*y*_0_) through minimization of the wavelet-domain Euclidean distance. Sub-pixel accuracy is obtained using the same paraboloid-fit refinement procedure as in WSVT to obtain (δ_*x*_, δ_*y*_).

A practical advantage of WXST over the conventional correlation method is that the DWT representation enables coefficient truncation, which reduces both noise and computational cost (Qiao, Shi, Celestre *et al.*, 2020 ▸). This benefit is especially significant for WXST because the template window must be large enough to include several speckle features to ensure uniqueness, producing 1D profiles that are typically much longer than those used in WSVT. As a result, the number of points involved in each pixel-by-pixel comparison is substantially higher, and the matching step can become time-consuming without dimensionality reduction.

Although each spatial window is flattened into a full-length 1D profile, the wavelet decomposition concentrates most of the relevant speckle information into a relatively small set of low- and mid-frequency coefficients. By discarding high-frequency detail coefficients that primarily contribute to noise, the dimensionality of the matching problem can be reduced substantially while retaining the information needed to uniquely identify the local speckle pattern. This truncated representation often improves the robustness and accuracy of the displacement estimate, particularly under low-contrast or low-signal conditions, and enables efficient two-image wavefront reconstruction.

The multi-resolution WXST algorithm (Qiao *et al.*, 2020 ▸) further accelerates the analysis by incorporating a coarse-to-fine search strategy. In this approach, the speckle images are downsampled through a multi-level image pyramid, and the displacement is first estimated on the most heavily downsampled (coarsest) level using a proportionally reduced search window. The coarse displacement map is then upsampled and used to define a much smaller, localized search window at the next finer level. Repeating this process across progressively finer scales significantly reduces the effective search window size at each stage while preserving the ability to capture large displacements introduced by strong phase gradients.

In the relative metrology module, the sample image must be compared with a separately measured reference image. In the absolute phase module, the experimentally measured speckle image is instead compared with a simulated speckle pattern computed from the coded-mask design and an idealized incident wavefront. This eliminates the need for a reference measurement and enables true single-shot wavefront sensing.

To generate the simulated reference image, the coded-mask pattern is propagated to the detector plane using a forward model that includes pattern generation and propagation, geometric magnification, detector sampling, pattern alignment, and partial-coherence blurring to match the experimental speckle scale (Shi *et al.*, 2025 ▸). Once the simulated reference is obtained, the WXST procedure proceeds exactly as in the relative mode. The resulting displacements represent absolute wavefront slopes relative to the idealized wavefront (*e.g.* a plane or spherical wave) used to generate the simulated speckle field.

### 2.3. SPINNet

SPINNet (Qiao *et al.*, 2022 ▸; Frith *et al.*, 2023 ▸) is a physics-informed convolutional neural network designed to infer speckle-pattern displacements directly from a pair of images, replacing the explicit wavelet-domain matching step used in WXST. The network takes as input a reference speckle image and a sample speckle image and outputs the transverse displacement fields δ_*x*_(*x*, *y*) and δ_*y*_(*x*, *y*). As in WXST, the underlying physical model assumes that the sample speckle pattern is a locally shifted version of the reference pattern, with the shifts being proportional to the corresponding wavefront gradients.

SPINNet consists of three main components: (1) a multi-scale feature extractor applied to both the sample and reference images, (2) a learned matching module based on a 3D cost volume that evaluates correlations between feature maps across candidate displacements, and (3) a refinement network that improves the accuracy and smoothness of the predicted displacement fields. The cost-volume construction directly mirrors the local search step used in traditional speckle tracking but replaces hand-crafted metrics with features explicitly optimized for speckle patterns. SPINNet employs a coarse-to-fine pyramidal architecture, analogous to the multi-resolution approach in WXST. This design allows the network to recover large displacements efficiently while maintaining high computational speed.

Because coded masks have known binary structures, large and diverse training datasets can be generated entirely through numerical simulation. Rather than relying on a single mask pattern, the SPINNet training set uses many different binary patterns and corresponding forward propagations to produce a broad distribution of simulated reference speckle images. Sample speckle images are then created by applying synthetic displacement fields drawn from physically motivated distributions. This enables the network to learn a generalizable mapping between speckle-pattern shifts and underlying phase gradients without requiring experimental ground-truth measurements, and ensures that the learned displacement estimator is consistent with the underlying wavefront-sensing physics.

Despite being trained exclusively on simulated data, SPINNet has demonstrated strong generalization to experimental coded-mask speckle images across variations in beam energy, mask pitch, detector geometry, and partial coherence conditions. The method can be used in either relative or absolute modes, since it only requires a reference–sample pair (measured or simulated). However, its displacement finding accuracy is generally lower than that obtained with WXST or WSVT. For this reason, SPINNet is typically used in *WFSuite* primarily for absolute-mode applications where single-shot operation, *in situ* compatibility, or rapid wavefront diagnostics are required, and where its two- to three-orders-of-magnitude speed advantage provides a practical benefit.

### 2.4. Post-process analysis across all methods

All three speckle-based methods, WSVT, WXST, and SPINNet, produce two transverse displacement fields, δ_*x*_(*x*, *y*) and δ_*y*_(*x*, *y*). These displacements are converted to phase gradients through the standard geometric relationship
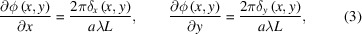
where λ is the wavelength, *L* is the propagation distance from the speckle generator (or sample if downstream) to the detector, and *a* is a geometry-dependent scaling factor (Qiao *et al.*, 2021 ▸; Shi, Highland *et al.*, 2023 ▸). These expressions produce two orthogonal gradient fields, ∂ϕ/∂*x* and ∂ϕ/∂*y*, defined at the detector plane.

The final wavefront or sample-induced phase map ϕ(*x*, *y*) is reconstructed by integrating these gradients using the Frankot–Chellappa algorithm (Frankot & Chellappa, 1988 ▸). This approach yields a smooth and globally consistent phase that is robust to local noise and small mismatches between the two gradient directions. For relative-mode measurements, the integrated phase map constitutes the primary output.

The effective spatial resolution of the reconstructed wavefront depends on the method. Although the phase maps are reported on a pixel grid, this does not necessarily imply pixel-limited spatial resolution. WSVT can approach detector-pixel-scale sensitivity when a sufficient number of speckle-generator positions and an adequate signal-to-noise ratio are available, whereas WXST uses a finite template window that acts as a spatial filter.

For absolute wavefront sensing, *WFSuite* performs an additional wavefront back-propagation step to recover the phase at the plane of the optical element or at the focal plane. Because WXST and SPINNet reconstruct the phase at the detector plane, the complex field is numerically propagated upstream or downstream using Fresnel propagation. As described by Shi *et al.* (2025 ▸), this enables accurate reconstruction of the wavefront at the optic or focal plane, providing a basis for quantifying aberrations, focal-spot quality, and nanofocusing performance.

## 3. Software design and implementation

**Figure 2:**
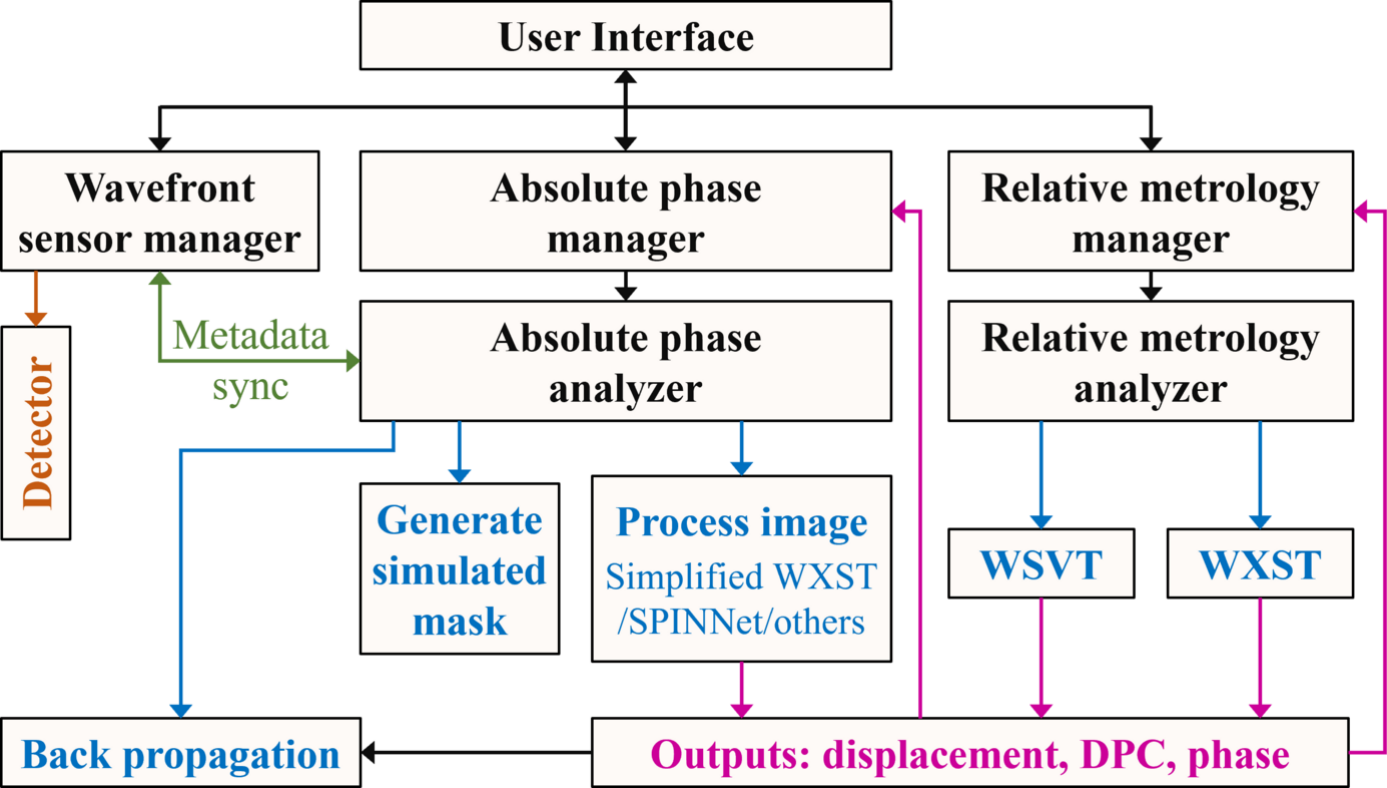
Software architecture of *WFSuite*.

*WFSuite* is implemented as a modular Python package that integrates detector acquisition, relative and absolute speckle-based wavefront sensing, neural-network inference, and wavefront back-propagation into a unified framework suitable for both real-time beamline diagnostics and offline analysis. Its architecture is shown in Fig. 2 ▸.

The general architecture of the software follows a Mediator pattern (Gamma *et al.*, 1995 ▸), leveraging ‘manager’ entities that separate the user interface from each business logic module, with the role of interpreting the commands of the user, organizing the input parameters, calling the proper functions, and collecting the output to be presented to the user (printouts, plots, *etc*).

At the top level, the user interface (graphic or command-line) provides the entry point for user operations and initializes the wavefront sensor manager, which handles detector configuration, acquisition control, file I/O, and experiment metadata such as pixel size, photon energy, and geometry. From the user interface, users may proceed to either the relative metrology or the absolute phase analysis pathways. The relative metrology analyzer implements the WSVT and WXST algorithms for multi-scan and two-image speckle tracking, while the absolute phase analyzer implements the coded-mask-based absolute wavefront-sensing workflow.

In particular, the absolute phase analysis first generates a simulated reference speckle pattern by propagating the known coded-mask structure to the detector plane. The measured speckle image is then compared with the simulated reference using either WXST or the SPINNet neural network to obtain single-shot displacement fields. Finally, the reconstructed phase at the detector plane is propagated upstream or downstream using *WOFRY*- (Sanchez del Rio *et al.*, 2024 ▸) or *SRW*-based (Chubar *et al.*, 2017 ▸) engines. The absolute phase manager can receive images directly from the sensor manager or operate on previously saved datasets, enabling both *in situ* diagnostics and offline reconstruction.

All analysis pathways converge to a standard output structure containing the transverse displacement fields, phase gradients, and integrated phase. In the absolute phase mode, these outputs are provided in the detector plane and can be numerically propagated to other locations. In the relative metrology mode, however, the outputs are provided in the plane of the downstream element, either the sample or speckle generator, depending on the experimental configuration.

*WFSuite* supports TIFF and HDF5 image formats and provides automated handling of cropping, rebinning, and I/O. All experiment and analysis parameters are stored in lightweight JSON configuration files, enabling reproducible workflows and efficient batch processing. Final results are saved in an HDF5 file with the reconstructed transmission, displacements, gradients, phase, and, when applicable, back-propagated intensity profiles.

The current version of *WFSuite* does not output statistical error maps for the reconstructed phase. The sub-pixel displacement search can provide high numerical precision, but this precision should not be interpreted as the total measurement uncertainty. In practice, the uncertainty is usually dominated by experimental factors, including photon statistics, speckle contrast, detector response, geometry calibration, and the quality of the mask or diffuser. When quantitative uncertainty estimates are required, users are encouraged to evaluate measurement repeatability using repeated measurements or reference regions. In absolute-mode measurements, systematic accuracy is additionally affected by coded-mask fabrication accuracy, detector response, and mask/detector alignment. *WFSuite* includes a calibration-path option in the absolute-mode workflow, allowing known systematic displacement offsets to be supplied and corrected during processing. However, determining such calibration maps is experiment-specific and beyond the scope of this software paper.

## 4. Applications and examples

To illustrate typical usage of *WFSuite*, we present two representative examples corresponding to the two major analysis pathways: (i) relative-mode metrology of a compound refractive lens (CRL) using WSVT, and (ii) absolute single-shot wavefront reconstruction of a focused beam using a coded mask, followed by numerical back-propagation to the focal plane. All examples reported in this section were run on a laptop PC with an Intel Core i9-11950H CPU and 32 GB RAM. The *WFSuite* source code, example datasets, and configuration files used in these examples are available via the links in the *Data availability* section. The GUI screenshots in this section illustrate the software interface and workflow; the detailed input parameters for reproducing the examples are provided in the corresponding configuration files.

### 4.1. Relative metrology of CRL using WSVT

Relative-mode measurements are widely used for the characterization of refractive X-ray optics, where the objective is to quantify the phase errors introduced by the optic, which directly correspond to its thickness variations. At the APS, we have established a standard procedure for characterizing transmission optics and have measured thousands of refractive lenses made from materials including Be, Al, diamond, Si, and polymer (Shi *et al.*, 2019 ▸, 2022 ▸; Shi, Highland *et al.*, 2023 ▸; Lin *et al.*, 2025 ▸).

**Figure 3:**
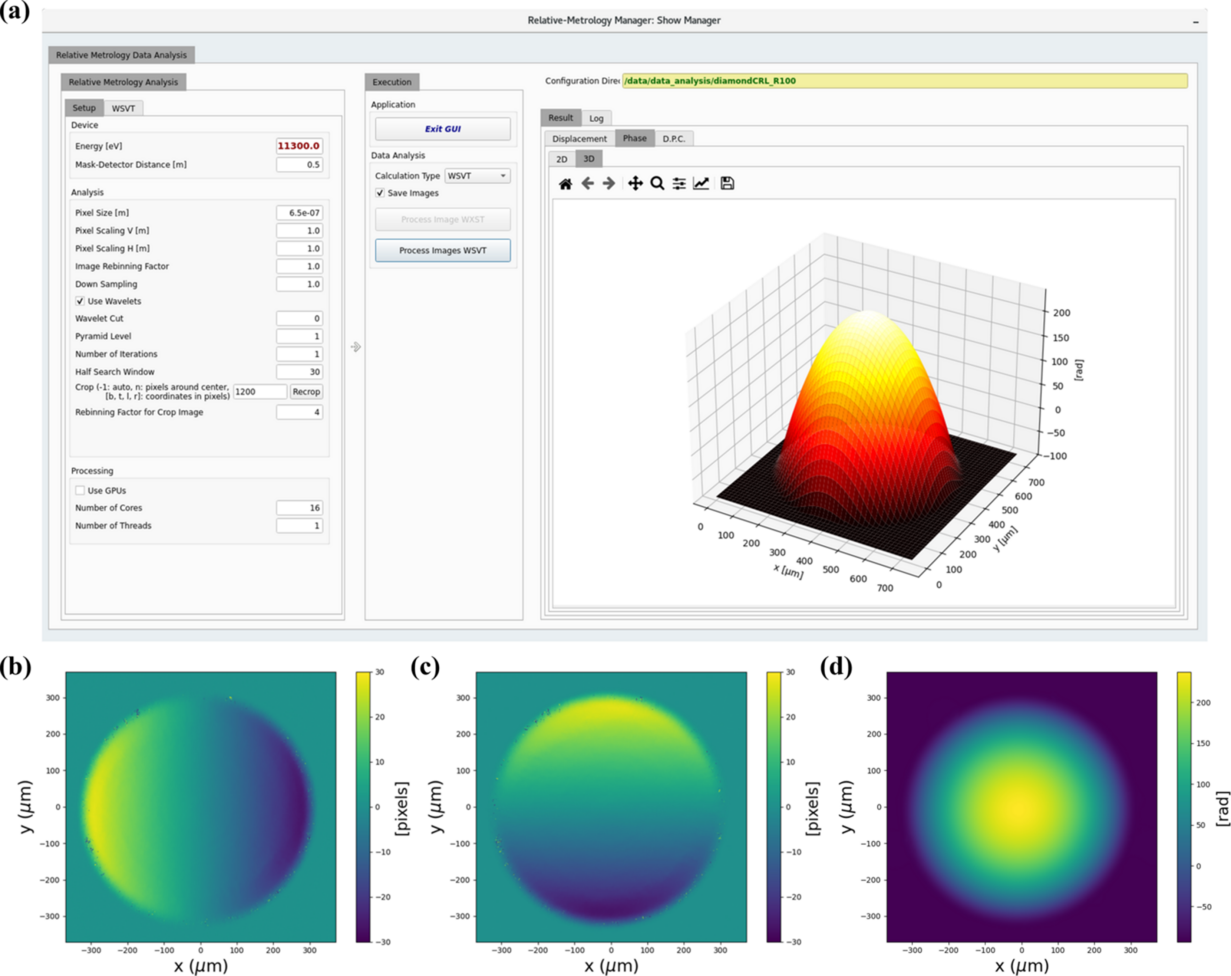
(*a*) Relative metrology manager GUI and reconstructed sample-induced phase. (*b*, *c*) Retrieved horizontal and vertical displacement fields. (*d*) Reconstructed 2D phase map.

An example analysis of a diamond lens using the WSVT method is shown in Fig. 3 ▸ to illustrate the relative metrology manager. The data used in this example were collected at a photon energy of 11.3 keV. The speckle pattern was generated by a coded mask placed upstream of the lens, consisting of a 2D binary random array of 2 µm Au squares with a 50% fill factor and a 1.5 µm thickness, and recorded 0.5 m downstream of the lens using an Andor Zyla camera coupled to a 10× objective, with an exposure time of 0.5 s per image. The GUI panel in Fig. 3 ▸(*a*) includes three blocks: input, execution, and output. Users may select either WSVT or WXST within the execution block, after which the corresponding input fields appear in the input block. Details of all parameters are provided in the operation manual distributed with the software.

Figs. 3 ▸(*b*) and 3 ▸(*c*) show the horizontal and vertical displacement fields obtained from WSVT. These fields represent the local speckle shifts between the reference and sample states and are converted to phase gradients using equation (3). The gradients are then integrated to recover the 2D phase map shown in Fig. 3 ▸(*d*), which directly reflects the thickness-induced phase error of the CRL. All reconstructed outputs are saved automatically to an HDF5 results file. Calculation parameters and basic phase statistics, including RMS and peak-to-valley values, are also saved in the resulting JSON output file. If the ‘Save Images’ checkbox in the execution block is enabled, *WFSuite* also automatically saves all generated figures to the specified output directory. Alternatively, individual plots can be saved manually using the save button in the output block, as shown in Figs. 3 ▸(*b*)–3(*d*). On the reference PC, the WSVT analysis shown here required approximately 50 s.

Depending on the optic or object under test, additional post-analysis steps are often required. For refractive focusing lenses, the reconstructed phase map is commonly converted to a thickness profile, and the ideal design curvature is removed to obtain the thickness-error distribution (Shi, Highland *et al.*, 2023 ▸). For crystals measured in relative mode, the interpretation and required processing differ substantially (Shi, Qiao *et al.*, 2023 ▸). Because these procedures depend strongly on the specific optic, material, and scientific goal, *WFSuite* does not enforce a fixed post-analysis workflow; instead, it leaves these steps to the end user.

### 4.2. Absolute single-shot wavefront sensing and focal-spot reconstruction

Absolute-phase-mode wavefront sensing uses a coded mask to replace the experimentally measured reference with a numerically simulated speckle pattern. This capability is particularly useful for characterizing focused beams and nanofocusing optics, where stable reference conditions may be difficult to obtain or where *in situ*, on-demand measurements are required. *WFSuite* implements this workflow through the Absolute Phase Manager, which integrates simulated-reference generation, phase retrieval using WXST or SPINNet, and optional numerical back-propagation to upstream or downstream planes.

**Figure 4:**
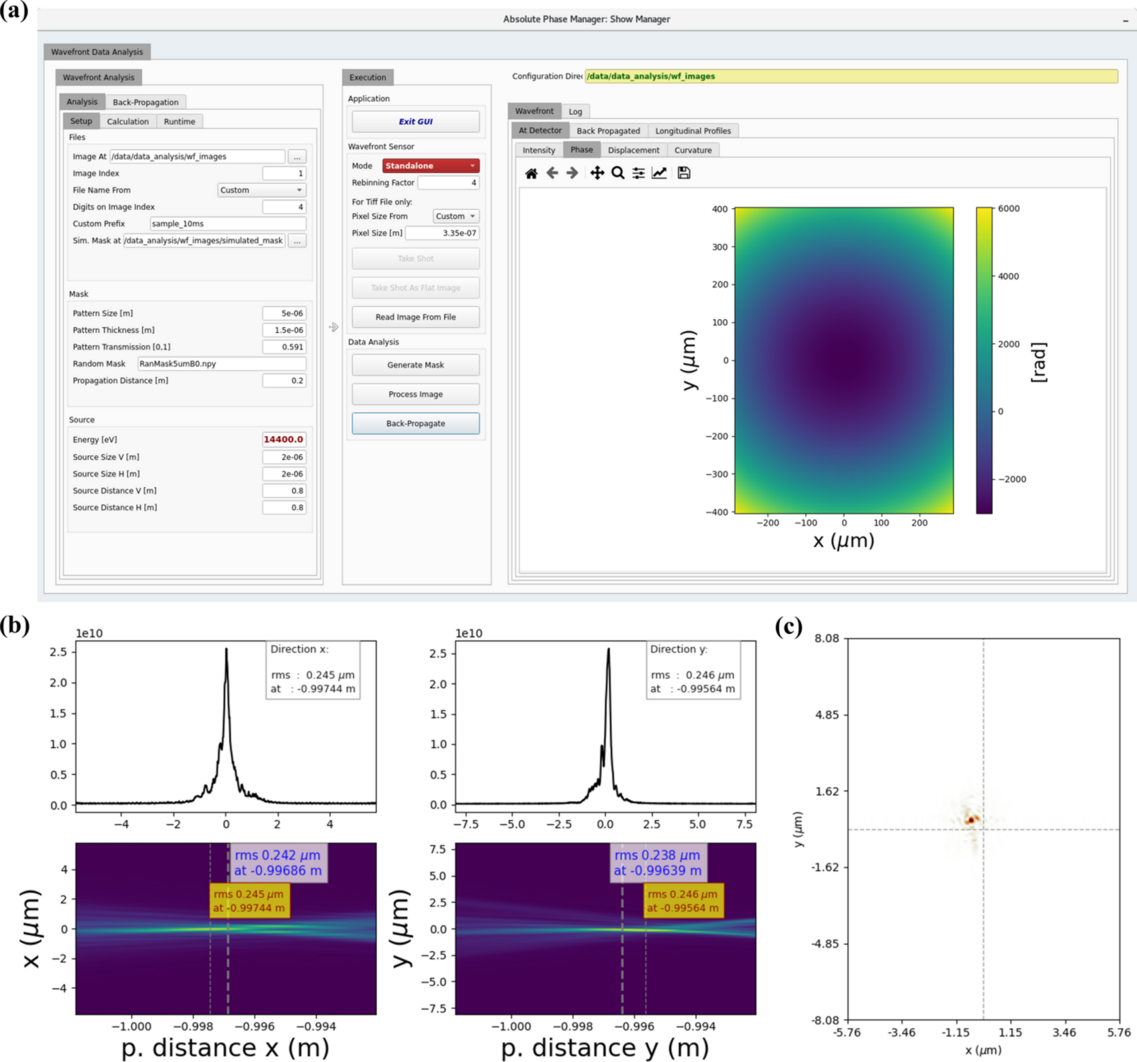
(*a*) Absolute phase manager GUI and reconstructed detector-plane phase. (*b*) Representative horizontal and vertical profiles of the back-propagated beam. (*c*) Reconstructed focal spot obtained by numerical back-propagation.

Fig. 4 ▸ illustrates an example measurement from a single-shot coded-mask image. The data used in this example were collected at a photon energy of 14.4 keV. The coded mask consisted of a 2D binary random array of 5 µm Au squares with a 50% fill factor and a thickness of 1.5 µm. The speckle image was recorded 0.2 m downstream of the mask using a Prosilica GT4400 camera coupled to a 10× objective, with an exposure time of 0.01 s. Fig. 4 ▸(*a*) shows the Absolute Phase Manager GUI with the input, execution, and output blocks. The reconstructed phase map at the detector plane is displayed within the output block. For this absolute-mode example, WXST required approximately 380 s on the reference PC, whereas the full SPINNet workflow took less than 5 s, with the neural-network inference itself accounting for only a small fraction of the total time.

After reconstructing the phase at the detector plane, *WFSuite* can numerically propagate the complex field upstream or downstream to recover the wavefront at physically relevant planes. For focusing optics, this includes propagation to the focal plane to visualize the focal-spot intensity and to quantify metrics such as spot size, symmetry, and astigmatism. Fig. 4 ▸(*b*) shows representative results from this back-propagation, including horizontal and vertical line profiles at various longitudinal positions. The output panel is interactive, allowing users to display line profiles at any propagation distance. Fig. 4 ▸(*c*) shows the corresponding 2D focal-spot intensity at the best-focus plane. The ability to obtain both detector-plane and focal-plane information from a single exposure is particularly valuable during beamline commissioning, alignment of nanofocusing optics, and real-time feedback optimization.

## 5. Conclusions

*WFSuite* provides a unified, modular platform for speckle-based X-ray wavefront sensing, supporting both relative measurements and single-shot absolute phase reconstruction using coded masks. Its integration of wavelet-based analysis, neural network inference, and numerical back-propagation enables flexible and reliable at-wavelength diagnostics for synchrotron and XFEL beamlines.

In practical use, WSVT is generally preferred for relative-mode analysis when multi-scan acquisition is available and the larger data volume is acceptable, because it provides higher robustness and sensitivity for quantitative optics metrology. For absolute-mode analysis, WXST provides higher quantitative accuracy, while SPINNet is preferred for rapid single-shot feedback or near-real-time operation.

Future developments will extend *WFSuite*’s capabilities in several directions. For relative-mode analysis, support for complementary speckle-based methods such as CMMI (Qiao *et al.*, 2021 ▸) will be added to enhance multicontrast imaging, including absorption, phase, and dark-field signals. In the absolute mode, additional SPINNet models trained on common beamline optics errors and realistic wavefront features will be incorporated, enabling optimized networks for beamline wavefront sensing. These models may also be combined with wavelet-based methods, with SPINNet providing an initial displacement estimate for WXST, allowing the search window and template size to be substantially reduced and improving computational efficiency. Optional post-processing modules, such as automated lens-thickness error analysis, will also be provided to streamline common metrology workflows. Finally, although the current software includes hardware integration for single-shot absolute measurements, future releases will introduce a more comprehensive relative-mode data-acquisition module to simplify multi-scan measurements.

## Data Availability

The *WFSuite* source code and documentation, including installation instructions and the operation manual, are openly available at https://github.com/APS-XSD-OPT-Group/WFSuite. Example datasets, configuration files, and supporting data required to reproduce the results are available at https://anl.box.com/v/WFSuite-example-data.
